# Genetic and Imaging Approaches Reveal Pro-Inflammatory and Immunoregulatory Roles of Mast Cells in Contact Hypersensitivity

**DOI:** 10.3389/fimmu.2018.01275

**Published:** 2018-06-05

**Authors:** Nicolas Gaudenzio, Thomas Marichal, Stephen J. Galli, Laurent L. Reber

**Affiliations:** ^1^Unité de Différenciation Epithéliale et Autoimmunité Rhumatoïde (UDEAR), UMR 1056, INSERM, Université de Toulouse, Toulouse, France; ^2^Laboratory of Cellular and Molecular Immunology, GIGA Institute, Liege University, Liège, Belgium; ^3^Faculty of Veterinary Medicine, Liege University, Liège, Belgium; ^4^WELBIO, Walloon Excellence in Life Sciences and Biotechnology, Wallonia, Belgium; ^5^Department of Pathology, Stanford University School of Medicine, Stanford, CA, United States; ^6^Department of Immunology and Microbiology, Stanford University School of Medicine, Stanford, CA, United States; ^7^Sean N. Parker Center for Allergy and Asthma Research, Stanford University School of Medicine, Stanford, CA, United States; ^8^Unit of Antibodies in Therapy and Pathology, INSERM Unit 1222, Department of Immunology, Institut Pasteur, Paris, France

**Keywords:** interleukin-10, tumor necrosis factor-alpha, avidin, two photon microscopy, mouse models, mast cells, contact hypersensitivity, IgE

## Abstract

Contact hypersensitivity (CHS) is a common T cell-mediated skin disease induced by epicutaneous sensitization to haptens. Mast cells (MCs) are widely deployed in the skin and can be activated during CHS responses to secrete diverse products, including some with pro-inflammatory and anti-inflammatory functions. Conflicting results have been obtained regarding pathogenic versus protective roles of MCs in CHS, and this has been attributed in part to the limitations of certain models for studying MC functions *in vivo*. This review discusses recent advances in the development and analysis of mouse models to investigate the roles of MCs and MC-associated products *in vivo*. Notably, fluorescent avidin-based two-photon imaging approaches enable *in vivo* selective labeling and simultaneous tracking of MC secretory granules (e.g., during MC degranulation) and MC gene activation by real-time longitudinal intravital microscopy in living mice. The combination of such genetic and imaging tools has shed new light on the controversial role played by MCs in mouse models of CHS. On the one hand, they can amplify CHS responses of mild severity while, on the other hand, can limit the inflammation and tissue injury associated with more severe or chronic models, in part by representing an initial source of the anti-inflammatory cytokine IL-10.

## Introduction

Allergic contact dermatitis (ACD), and its animal model contact hypersensitivity (CHS), are T cell-mediated skin inflammatory diseases caused by delayed-type hypersensitivity responses to environmental allergens (1–3). An important group of contact allergens are small organic compounds that rapidly penetrate into the skin and bind to proteins in the dermis, a process called ‘‘haptenization’’ that alters self-proteins and renders them antigenic. Sensitization to haptens is characterized by the activation of dendritic cells (DCs), which migrate to the skin-draining lymph nodes, and by the priming of allergen specific T cells. Re-exposure of the skin to the same hapten leads to development of a type of delayed-type hypersensitivity reaction to that antigen mediated by T cells (3). Besides T cells, other immune cell populations, including neutrophils (4), natural killer (NK) cells (5), and innate lymphoid cells (6), can influence the magnitude and duration of CHS responses. This can be particularly important during severe CHS reactions, in which the immune system must dampen the intensity of the pathology in order to maintain optimal tissue integrity.

Mast cells (MCs) are widely deployed in many tissues, including the skin, and can represent powerful sentinels of the immune system (7–10). MCs can be stimulated *via* the high-affinity receptor for IgE (FcεRI), or by any of multiple other mechanisms [including activation by the KIT ligand stem cell factor (SCF), immune complexes of IgG, various complement peptides, cytokines and chemokines], leading to the release a diverse spectrum of biologically active mediators, including some with pro- or anti-inflammatory functions (9, 11). As a result, MCs can have potentially important effector or immunoregulatory functions during inflammatory processes, including during the sensitization and effector phases of CHS responses.

Different sophisticated mouse models and fluorescent avidin-based imaging tools can now be used to study MC functions *in vivo* and to visualize the dynamics of MCs, the release of MC granules, and MC gene activation using intravital two-photon microscopy. The combined use of such genetic and imaging tools has shed new light on how skin MCs, on the one hand, can amplify CHS responses of mild severity while, on the other hand, can limit the inflammation and tissue injury associated with more severe or chronic models, in part by representing an initial source of the anti-inflammatory cytokine IL-10.

## Mouse Models to Investigate the Roles of MCs and Mast Cell-Associated Products *In Vivo*

In order to investigate the potential contributions of MCs or MC products in particular biological settings, the “holy grail” would be to be able to solely and selectively deplete MCs or MC products *in vivo* (12, 13). Enormous progress has been made to reach this goal since the discovery by Kitamura and colleagues that WBB6_F1_-*Kit^W^/Kit^W-v^* mice, hereafter named *Kit^W/W-v^* mice, were profoundly deficient in MCs (14). *Kit^W/W-v^* and C57BL/6-*Kit^W-sh^/Kit^W-sh^* mice, hereafter named *Kit^W-sh/W-sh^* mice, are the two most common strains of MC-deficient mice with abnormalities affecting KIT, the receptor for the main MC growth and survival factor, SCF (15, 16). These mice are also generally called “*Kit* mutant” mice. *Kit*-mutant mice not only exhibit a profound MC deficiency but also a variety of other phenotypic abnormalities and have been widely used to analyze the functions of MCs *in vivo* (11, 17–20). Since differences in the biological responses in *Kit* mutant mice compared with wild-type (WT) mice may not be solely due to their deficiency in MCs, we and others have used “MC *knock-in* mice” to assess the importance of MCs in regulating the expression of biological responses in *Kit*-mutant mice. Such an approach consists of restoring the MC deficiency in *Kit*-mutant mice by adoptively transferring genetically compatible, *in vitro*-derived WT or mutant MCs (10, 18, 19, 21, 22). This approach can be helpful, but it may not be possible to prove that such adoptively transferred MCs are fully identical (in either anatomical location, phenotype, or function) to those in the same anatomical location as in the corresponding WT mice (13, 18, 23).

In addition to these *Kit*-mutant mice, newer models in which the MC deficiency is not dependent on mutations affecting the structure or expression of KIT have emerged (Table 1). They consist of genetically modified mice that exhibit constitutive deficiencies (*Cpa3^Cre/+^*, *Cpa3-Cre*^+^; *Mcl-1^fl/fl^*, *Mcpt5-Cre*^+^; *DTA^fl/+^*) or diphtheria toxin-inducible deficiencies [*Mcpt5-Cre*; *iDTR^fl/+^*, “Mas-TRECK,” “RMB” (these mice are described in detail in the section entitled The Red Mast cell and Basophil mouse)] in populations of MCs. In some of such models, there are also abnormalities in numbers and functions of basophils (24–29).

**Table 1:** Genetic and imaging approaches to study mast cell (MC) functions *in vivo*.

A—Mouse models to investigate the roles of MCs*in vivo*				
Names	MC deficiency	Reference [reviewed in Ref. (9, 10, 20)]		
WBB6_F1_-*Kit^W^/Kit^W-v^*	Constitutive	(11, 14)		
C57BL/6-*Kit^W-sh^/Kit^W-sh^*	Constitutive	(15)		
Cre-MASTER *Cpa3^Cre/+^*	Constitutive	(22)		
Hello *KittyCpa3-Cre^+^*; *Mcl-1^fl/fl^*	Constitutive	(23)		
*Mcpt5-Cre^+^*; *DTA^fl/+^*	Constitutive	(21)		
*Mcpt5-Cre^+^; Gata2^fl/fl^*	Constitutive	(31)		
*Mcpt5-Cre*; *DTR^fl/+^*	Inducible	(21)		
Mas-TRECK	Inducible	(24)		
Red MC and Basophil (RMB)	Inducible	(26)		

**B—Genetically encoded fluorescent tracers to visualize MCs*in vivo***				

**Names**	**Fuorescent tracers**	**Reference**		

*Mcpt5-Cre*; *ROSA26-EYFP*	EYFP [Ex_max_ = 513 nm/Em_max_ = 527 nm]	(21, 30, 35, 36)		
*Mcpt5-Cre*; Ai6	ZsGreen [Ex_max_ = 496 nm/Em_max_ = 506 nm]	(38)		
*Mcpt5-Cre; R26-td-RFP*	td-RFP [Ex_max_ = 555 nm/Em_max_ = 584 nm]	(40, 41)		
RMB	d-tomato [Ex_max_ = 554 nm/Em_max_ = 581 nm]	(26)		

**C—Non-genetic approaches to visualize skin MC secretory granules***in vivo*****				

**Fluorescent tracers**	**Injection (site)**	**Injection (timing)**	**Staining**	**Reference**

Av.SRho [Ex_max_ = 512 nm/Em_max_ = 525 nm]	Intradermal	10–15 min before analysis	Exteriorized MC granules	(35)
Av.SRho [Ex_max_ = 512 nm/Em_max_ = 525 nm]	Intradermal	7 days before analysis	Intracellular MC granules	(36)

As reviewed in detail elsewhere (12, 13, 23), each model currently available has limitations that must be kept in mind when interpreting the results of studies using such mice, and the importance of particular MC (or basophil) roles in individual biological responses may vary both according to the details of the model used to study that biological response and based on the strain background of the mice. Analyzing to what extent MC-associated products contribute to a particular biological response requires that the gene coding for such molecule would be specifically deleted/inactivated in MCs. In this regard, “MC-specific CRE” mice, which express CRE recombinase under the control of a promoter specific for MCs (26, 30–33), may allow for specific deletion of floxed genes in MCs. Interestingly, Li et al. recently used this approach to delete the transcription factor GATA2 in connective tissue-type MCs (CTMCs) by crossing *Gata2^fl/fl^* mice with *Mcpt5-Cre* mice [which express the CRE recombinase in connective tissue MCs (these mice are discussed in detail in part 4.1, below)] (34). The authors found that *Mcpt5-Cre^+^; Gata2^fl/fl^* mice exhibit a nearly complete deficiency in CTMCs in the skin, stomach, trachea, and peritoneal cavity, while having comparable number of basophils, T cells, B cells, NK cells, neutrophils, and macrophages (34). These mice thus represent another useful model of constitutive deficiency in CTMCs.

## The Use of Genetically Encoded Fluorescent Tracers to Visualize MCs *In Vivo*

### The Mcpt5 Cre/loxP Reporter System

The *Mcpt5* gene encodes the mouse MC protease 5, also known as α-chymase, that is predominantly detected in connective tissue MCs [i.e., mostly skin and peritoneal MCs (PMCs)] (35). In 2010, Scholten and coworkers reported the generation of the *Mcpt5-Cre* mouse strain in which a modified *iCre* gene [i.e., encoding an improved CRE recombinase (36)] cassette was strategically inserted into the *Mcpt5* gene (33). Importantly, compared to the *Cpa3-Cre* mouse strain reported by Feyerabend et al. in 2011 in which the targeted insertion of *iCre* gene into the carboxypeptidase A3 (*Cpa3*) locus deleted MCs *via* a genotoxic mechanism (25), the *Mcpt5-Cre* mouse did not show any signs of CRE-mediated genotoxicity. The *Mcpt5-Cre* mice were bred with a ROSA26_Enhanced Yellow Fluorescent Protein (EYFP) reporter strain, in which the gene encoding EYFP [a yellow fluorescent tracer (Ex_max_ = 513 nm/Em_max_ = 527 nm)] has been placed under the control of the ubiquitous ROSA26 promoter flanked by loxP stop elements (37). The resulting *Mcpt5-Cre*; *ROSA26-EYFP*, thereafter named Mcpt5-EYFP, double transgenic mice expressed EYFP fluorescence signal specifically in peritoneal and skin MCs (33). These Mcpt5-EYFP mice then were used in different studies by our (38, 39) and other (24) groups in order to monitor the behavior of dermal MCs during inflammatory reactions using intravital two-photon systems in living mice. As a first example of such an application, Dudeck et al. used the Mcpt5-EYFP mice to visualize and monitor simultaneously perivascular EYFP^+^ dermal MCs and Qdot-labeled blood vessels by intravital two-photon microscopy in a model of moderate CHS. Upon induction of the disease, they reported that EYFP^+^ perivascular MCs exhibited changes in their morphology in parallel with the dilatation of blood vessels and vascular leakage of Qdots into the dermis (24).

*Mcpt5-Cre* mice were also bred with *ROSA26-ZsGreen* mice (40) [i.e., also called Ai6 mice, expressing the *Zoanthus* sp. Green fluorescent protein (Ex_max_ = 496 nm/Em_max_ = 506 nm) with a targeted insertion of a construct containing the strong and ubiquitous CAG promoter in the ROSA26 locus] (41). Compared to the previously described *ROSA-EYFP* mouse, the Ai6 mouse has been reported to express a stronger fluorescence signal and is thought to be more appropriate to visualize discrete cellular projections *in vivo*. Using the *Mcpt5-Cre;* Ai6 double transgenic mice and intravital two-photon microscopy, the authors showed that ZsGreen^+^ skin MCs can “sample” circulating IgE by extending cell processes across the vessel wall (41).

Recently, Dudeck and colleagues mated *Mcpt5-Cre* mice with *R26-td-RFP* reporter mice (42), i.e., expressing the tandem dimer_Red Fluorescent Protein [Ex_max_ = 555 nm/Em_max_ = 584 nm], under the control of the ROSA26 promotor. The resulting *Mcpt5-Cre; R26-td-RFP*, also called Mcpt5-RFP, double transgenic mice were expressing the RFP specifically in peritoneal and skin MCs (43). In this study, the authors then bred the Mcpt5-RFP mice with the DC reporter strain *Cd11c-EGFP* (44), i.e., expressing the Enhanced Green Fluorescent Protein [Ex_max_ = 488 nm/Em_max_ = 509 nm] under the control of the *Cd11c* gene promotor, and used the *Mcpt5-RFP; Cd11c-EGFP* triple transgenic mice to track simultaneously RFP^+^ MCs and EGFP^+^ DCs (the emission wavelengths of RFP and EGFP fluorescence being far enough apart to be analyzed simultaneously) in the skin of living mice. Using such conditions, they reported that MCs and DCs can exchange membrane proteins in a model of moderate hapten-induced CHS, as discussed in more detail in the section Pro-inflammatory functions of MCs in CHS (43). We will discuss below a potential alternative to the time-consuming and costly approach consisting of the generation of double or triple transgenic animals. This approach permits labeling and monitoring of exteriorized or intracellular skin MC granules using intravital imaging.

### The Red MC and Basophil Mouse

In 2014, Dahdah and coworkers described a new *knock-in* mouse model named the “Red MC and Basophil” (RMB) mouse (29). The RMB mouse is based on the insertion of a cassette composed of an internal ribosomal entry site, a sequence coding for the protein red tandem dimer-Tomato, i.e., tdT, a genetically encoded red fluorescent tracer [Ex_max_ = 554 nm/Em_max_ = 581 nm] (45), a 2A cleavage sequence, and the human diphtheria toxin receptor (DTR) in the 3′-UTR of the *Ms4a2* gene [encoding the FcεRIβ chain that is reported to be specifically expressed in MCs and basophils (46, 47)]. This mouse model allows the tracking of MCs and basophils based on their expression of tdT red fluorescence and render both cell types vulnerable to diphtheria toxin treatment. Using *ex vivo* flow cytometry, the authors reported that FcεRI^+^KIT^+^ PMCs and FcεRI^+^KIT^-^CD49b^+^ blood and splenic basophils were highly positive for the tdT and that two intraperitoneal injections of diphtheria toxin were sufficient to deplete both cell types, including FcεRI^+^KIT^+^ skin MCs. Importantly, diphtheria toxin treatment of RMB mice apparently did not affect other tested blood cell populations, including red blood cells, CD19^+^ B cells, CD4^+^ and CD8^+^ T cells, neutrophils, eosinophils, and monocytes (29). They also showed that tdT^+^ MCs and basophils exhibited distinct kinetics of repopulation in tissue upon diphtheria toxin treatment. While the percentage of blood tdT^+^ basophils was back to normal at day 12 posttreatment, only 50% of the tdT^+^ PMC population was restored at 6 months posttreatment. Therefore, the RMB mouse could be used either as a model of both basophil- and MC-deficient mouse when used at day 6 posttreatment with diphtheria toxin, or as a model of basophil-sufficient but MC-deficient mouse when used at day 12 posttreatment. In this study, the authors reported that MCs were detrimental to survival in a model of severe sepsis by impairing peritoneal macrophages phagocytosis (29). Finally, this model could also potentially be used to monitor either basophil or MC behavior in different tissues, including the skin, using intravital imaging microscopy.

## The Use of New Non-Genetic Approaches to Visualize MC Secretory Granules *In Vivo*

### Dynamic Analysis of Exteriorized MC Granules *In Vitro* and *In Vivo*

A principal characteristic of MCs is their capacity to respond to a broad panel of activation signals and rapidly exteriorize intracellular secretory granules enriched in pre-stored bioactive molecules, e.g., histamine, proteases, TNF, etc., during inflammatory reactions and influence ongoing immune response (7, 9, 48, 49). Although important progress has been made in efforts to localize and track MCs *in vivo*, i.e., Cre/LoxP or RMB reporter mice, the lack of *in vivo* MC granule-specific tracking systems has limited the analysis and the understanding of the dynamics and quantitative characteristics of MC granule exteriorization strategies.

**Figure 1:**
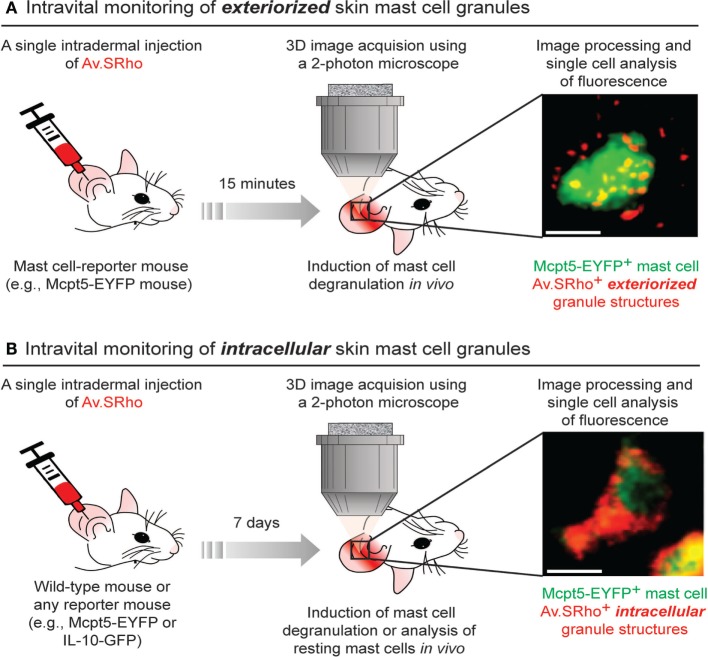
Fluorochrome-labeled avidin can be used to probe either exteriorized or intracellular mast cell (MC) granule structures in living mice. **(A)** 5 µg of sulforhodamine 101-coupled avidin (Av.SRho) is injected intradermally (i.d.) into the ear pinna of a MC-reporter mouse (e.g., Mcpt5-EYFP). 15 min later, the mouse is anesthetized and placed under a two-photon microscope; MC degranulation is induced by i.d. injection of stimulus; 3D high-resolution images of single cells are taken and Av.SRho fluorescence signal is assessed. The photograph shows a single dermal EYFP^+^ MC activated upon i.d. injection of substance P exhibiting Av.SRho**^+^** exteriorized small granule structures. Image extracted from Gaudenzio et al. (38). **(B)** 5 µg of Av.SRho is injected i.d. into the ear pinna of a mouse. 7 days later, the mouse is anesthetized and placed under a two-photon microscope; 3D high-resolution single-cell images are taken and Av.SRho fluorescence signal is assessed. The photograph shows a single EYFP^+^ MC exhibiting Av.SRho^+^ intracellular granule structures. Image extracted from Reber et al. (39).

To circumvent those constraints, we developed a new approach that permits the monitoring, in real-time and at the single-cell level, of the spatially complex, rapidly evolving features of MCs undergoing activation using both real-time confocal microscopy *in vitro* or non-invasive two-photon microscopy in living mice (38, 50) (Figure 1A). The matrix of MC granules is composed of abundant proteoglycans, in CTMC consisting mostly of heparin and its core peptide serglycin, a highly anionic macromolecular complex in which MC bioactive mediators are embedded (51, 52). Fluorochrome-labeled avidin [a cationic molecule with a very high-affinity for heparin (53)] can be used to detect exteriorized MC granule structures *in vitro* and *in vivo* (38, 50, 54). As an example of the *in vivo* application of this method, we found that substance P [predominately *via* the MC receptor for cationic molecules MrgprB2 (55)] induced dermal MCs rapidly to secrete small and relatively spherical granule structures, a pattern consistent with the secretion of individual granules (38). Conversely, activating dermal MCs with IgE/antigen (*via* FcεRI) increased the time partition between signaling and secretion, which was associated with the formation of larger and more heterogeneously shaped granule structures that underwent prolonged exteriorization. IgE/antigen- and substance P-dependent activation *in vivo* were also associated with distinct local and systemic pathophysiological responses (38).

### Using Fluorochrome-Labeled Avidin to Probe Intracellular MC Granules in Living Mice

We next investigated whether we could also use fluorochrome-labeled avidin to specifically label intracellular MC granules *in vivo* and use this probe as a non-genetically encoded MC tracer in the skin of living mice. To this end, we injected fluorochrome-labeled avidin into the ear dermis of mice and analyzed the location and intensity of the avidin fluorescence signal 7 days after the injection. Surprisingly, we found that fluorochrome-labeled avidin was located, apparently nearly exclusively, within dermal MC intracellular secretory granules and that such “fluorescent MC granules” could be visualized for many weeks in the same animal using longitudinal intravital two-photon microscopy (Figure 1B).

Therefore, this approach can be employed regardless of the mouse genetic background, as long as the studied mouse strain exhibits negatively charged proteoglycans in MC granules, and enables the direct visualization of skin MC granule content for a prolonged period of time. If combined with a reporter strain, it also permits the longitudinal monitoring of gene activation in such labeled MCs in living mice (39) (Figure 1B).

## Role of MCs in CHS: A Long Controversy

The potential role of MCs in CHS has been assessed for almost 30 years, and most of the results found in the literature might make one conclude that their actual contribution remains to be clarified. Indeed, the use of different MC-deficient animals in several experimental conditions has suggested either a positive immunomodulatory role (24, 27, 39, 56–60), a negative immunomodulatory role (24, 39, 60–63), or no contribution (64–66) for MCs in the CHS models and conditions tested.

Although the reasons for such discrepant results are not yet fully understood, it is clear that the outcome of the CHS response and the subsequent interpretation of the results might be influenced by numerous factors, including the type of mutant MC-deficient mice, hapten and protocol tested. Last but not least, unreported and more unpredictable variables can come from the animals, e.g., gender, age, and microbiome/pathogen status, and the housing conditions. Several studies indeed suggest that the commensal microbiota may substantially influence both the immune homeostasis and the behavior of particular hematopoietic cells (67–69).

Dudeck et al. first assessed the influence of the type of MC-deficient mice used (24). Consistent with a previous report (61), they observed that DNFB-sensitized *Kit^W-sh/W-sh^* and *Kit^W/W-v^* MC-deficient mice exhibited enhanced ear swelling after DNFB challenge. However, they found that, in the same model, the ear swelling was significantly reduced when MCs were depleted by injection of DT in *Mcpt5-Cre; iDTR* mice (24). Since *Kit* mutant mice have many phenotypical abnormalities beside their MC deficiency (18, 20, 70–73), the authors concluded that the exaggerated CHS responses in these mice is not caused by the absence of MCs but is related to the KIT deficiency (24). In striking contrast with this, we recently reported that both *Kit^W-sh/W-sh^* mice and the *Kit*-independent *Cpa3-cre^+^; Mcl1^fl/fl^;* and *Mcpt5-Cre^+^; DTA* MC-deficient strains have significantly enhanced ear swelling and epidermal hyperplasia compared to the values in their respective littermate controls in a severe CHS model induced by DNFB (39). However, the *Kit^W-sh/W-sh^* mice exhibited a more pronounced increase in ear swelling in this model as compared to *Kit*-independent MC-deficient mice.

Using a chronic model of oxazolone-induced CHS, Gimenez-Rivera and colleagues also recently reported increased CHS responses in both *Kit^W-sh/W-sh^* mice and *Kit*-independent *Mcpt5-Cre^+^; iDTR* MC-deficient mice (63). Similarly to us, the authors noticed that the responses were more pronounced in *Kit^W-sh/W-sh^* mice, since these mice but not the *Kit*-independent strain, showed skin scaling and necrosis at the site of chronic CHS reactions (63). We think that the simplest interpretation of these results is that MCs can have effects that can significantly limit the assessed features of these severe and chronic CHS models in each of the examined mouse strains, but that additional phenotypic abnormalities in *Kit^W-sh/W-sh^* mice, beside their MC deficiency, probably also contribute to the exacerbation of severe CHS responses in this strain, as suggested by Dudeck et al. (24).

The influence of the dose of haptens used for sensitization and/or challenge was studied in detail in *Kit^W/W-v^* mice (60). The authors reported that the ear swelling and the recruitment of leukocytes was reduced in *Kit^W/W-v^* mice as compared to *Kit^+/+^* mice in a CHS model consisting of sensitization with a low dose of oxazolone, and challenge with the same hapten. These values were restored to levels observed in *Kit^+/+^* mice after intradermal engraftment of bone marrow-derived cultured MCs (i.e., BMCMCs) into the ear pinnae of *Kit^W/W-v^* mice (60). However, when using a higher dose of oxazolone for the sensitization, the authors found opposite results, i.e., significantly increased ear swelling in MC-deficient *Kit^W/W-v^* mice as compared to both *Kit^+/+^* mice and *Kit^W/W-v^* mice engrafted with BMCMCs (60). These results suggest that, depending on the severity of the CHS model used, MCs can have either a positive or a negative immunomodulatory role. We recently confirmed this using a different hapten (DNFB) and *Kit*-independent *Cpa3-cre^+^; Mcl1^fl/fl^* MC-deficient mice, indicating that the plasticity of MC responses in CHS models is probably not due solely to KIT-related phenotypic abnormalities and does not depend on the type of hapten used (39).

## Potential Mechanisms of MC Activation in CHS

**Figure 2:**
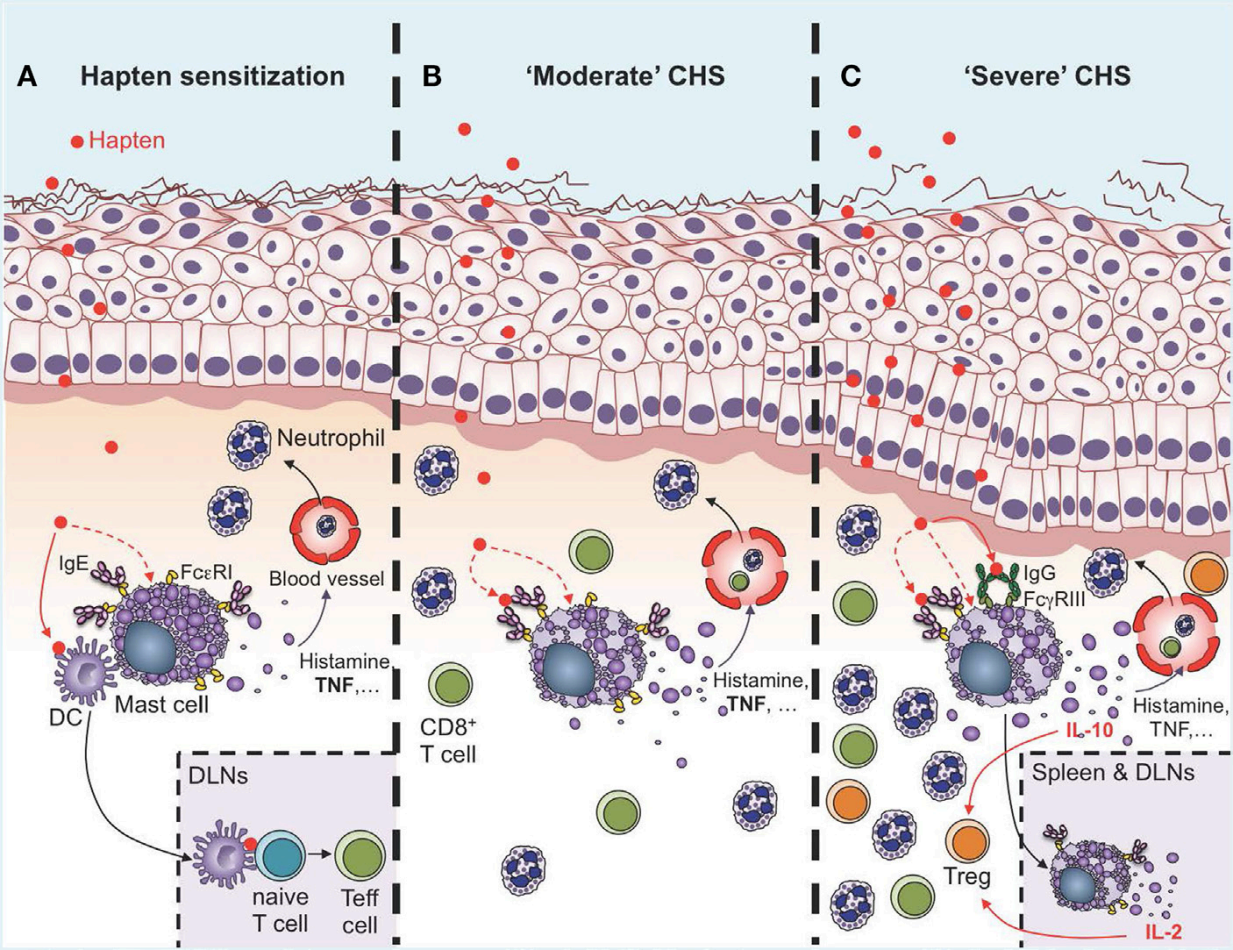
Roles of mast cells (MCs) in the sensitization and effector phases of contact hypersensitivity (CHS). **(A)** During the sensitization phase, MCs are activated directly or indirectly by haptens to release of a diverse spectrum of mediators, including histamine and TNF, which induce vasodilatation and recruitment of leukocytes, mainly neutrophils. Multiple lines of evidence suggest that IgE can amplify MC activation in the sensitization phase though antigen-independent cytokinergic effects. Direct cell-to-cell contacts between MCs and dendritic cells (DCs), as well as MC-derived TNF, can amplify DC migration to the draining lymph nodes (DLNs), where these cells prime naïve T cells to become effector cells (Teff cells) *via* antigen presentation. **(B)** During “moderate” CHS responses, MCs and MC-derived TNF amplify ear swelling, leukocyte recruitment (mainly neutrophils and CD8^+^ T cells), and epidermal hyperplasia. Evidence suggests that IgE and FcεRI can amplify the pro-inflammatory functions of MCs in moderate CHS **(C)**. During more severe (and chronic) CHS responses, MCs represent an early source of IL-10 in the skin, which amplifies recruitment of regulatory T cells (Treg) and limits ear swelling and epidermal hyperplasia. MC activation in the skin is amplified by the engagement of FcγRIII by hapten-IgG immune complexes. Additionally, MCs can migrate to the DLNs and the spleen in an IgE-dependent manner, where they produce IL-2 which helps in maintaining the Teff:Treg ratio at the site of inflammation, and thereby contributes to limiting the severity of CHS responses.

Multiple lines of evidence indicate that skin MCs are activated both during the sensitization and challenge phases in CHS models (Figure 2). Skin exposure to haptens induces innate immune responses which could directly or indirectly activate MCs. Indeed, Dudeck et al. reported MC degranulation in skin sections obtained 4 h after sensitization with DNFB (59). A recent report also shows that DNFB, but not oxazolone, can induce direct degranulation of rat PMCs *in vitro* (74). MC activation has been best visualized in the effector phase using two-photon microscopy. First, Dudeck et al. used Mcpt5-EYFP reporter mice to demonstrate that perivascular MCs exhibit important changes in their morphology upon hapten challenge (24). More recently, we precisely monitored MC degranulation in a severe DNFB-induced CHS models using fluorescently labeled avidin to stain MC intracellular granules (39). Before DNFB challenge, only ~10% of dermal MCs were marginally degranulated, exhibiting just a few fluorescent avidin^+^ granules outside of the cell. However, as early as 1 day after DNFB challenge, ~60% of dermal MCs were extensively degranulated and this number reached ~90% at day 2 (39).

How MCs are activated upon challenge in CHS models is not fully understood. One possibility could be that, similarly to what is observed during immediate hypersensitivity reactions in the skin, activation of MCs in CHS could require crosslinking of antigen-specific IgE bound to FcεRI on the surface of MCs. Indeed, mice deficient for IgE have reduced ear swelling and leukocyte infiltration in models of CHS to oxazolone or DNFB (58). Surprisingly however, contact sensitivity to oxazolone in *IgE*^−/−^ mice could be restored by administration of hapten-irrelevant IgE before sensitization (58). The authors obtained similar results after transfer of four different IgE clones directed against dinitrophenyl or trinitrophenyl. Interestingly, two of these IgE clones (SPE-7 and C38-2) were previously shown to be cytokinergic, that is, to induce the release of cytokines in MCs in an antigen-independent manner (75, 76). Bryce and colleagues found that exposure to oxazolone upregulates mRNA levels for the MC-specific gene *Mcpt6*, and for genes encoding several cytokines and chemokines required for efficient sensitization. mRNA levels for most of these genes, including *Mcpt6*, were reduced in oxazolone-treated *IgE*^−/−^ mice, and restored by prior administration of IgE of irrelevant specificity (58). Taken together, these results suggest that IgE can control sensitization though antigen-independent cytokinergic effects on MCs in this CHS model (58) (Figure 2A). More recently, Kobayashi and colleagues also reported reduced ear swelling and leukocyte infiltration in mice deficient for the β chain of FcεRI in a CHS model to oxazolone (77). In the same model, administration of a recombinant soluble form of human FcεRI, which binds both human and mouse IgE, after oxazolone challenge also significantly reduced CHS responses, suggesting that IgE could amplify MC activation in the effector phase of this CHS model (77).

Mast cells express the activating IgG receptor FcγRIII and could thus also be activated by IgG immune complexes during CHS responses. Evidence of this has been reported by Grimbaldeston et al. in a model of severe CHS, and will be discussed below in the section Pro-inflammatory functions of MCs in CHS in more detail (61). However, it should be noted that whether, and in specifically what circumstances, antibodies (IgE or IgG) play a significant role in CHS is still a matter of debate. First, CHS is largely considered to be mediated by CD8^+^ T cells, which are primed in lymphoid organs during the sensitization phase and recruited in the skin upon re-exposure with the hapten (3). Second, in 2006, O’Leary et al. reported normal CHS responses to DNFB and oxazolone in two types of T cell- and B cell-deficient mice, i.e., *Rag2*^−/−^ and SCID mice (5). CHS reactions in these T and B cell-deficient mice still required a sensitization phase, and the authors discovered that such antibody-independent adaptive immune response to haptens was mediated by NK cells (5).

## Pro-Inflammatory Functions of MCs in CHS

Data obtained by independent groups using both *Kit* mutant and *Kit*-independent MC-deficient mice have confirmed that MCs can have important pro-inflammatory functions in CHS models of moderate severity (24, 27, 39, 56–60) (Figure 2B). MCs can amplify neutrophil recruitment at the site of hapten exposure both during the sensitization phase (4), and after challenge (24, 57). Interestingly, recruitment of neutrophils seems to be a key step for hapten sensitization, and neutrophil-depleted mice exhibit decreased migration of DCs to the local draining lymph nodes (DLNs) and reduced contact allergen-induced T cell priming (4). Recent evidence indicates that MCs might also interact directly with DCs during CHS responses. Dudeck et al. used *Mcpt5-RFP; Cd11c-EGFP* triple transgenic mice to track simultaneously RFP^+^ MCs and EGFP^+^ DCs in the skin of living mice during CHS responses using intravital two-photon microscopy (43). Using this approach, they observed targeted cell-to-cell interactions between MCs and DCs. Skin DCs dynamically “scanned” MCs at early time-points of CHS responses, while at a later stage, long-lasting MC–DC interactions occurred, with evidence of DC-to-MC molecule transfers, including major histocompatibility complex class II (MHCII) proteins. The authors also provided evidence that such transfer of MHCII-conferred antigen-presenting capability to MCs enabled MCs to prime allogenic T cells in this model of moderate CHS (43).

While direct cell-to-cell contacts likely are involved in the pro-inflammatory functions of MCs in CHS, as suggested in this study (43), soluble factors released by MCs might also potentiate the inflammatory response and leukocyte recruitment in CHS. Among these, TNF is a key mediator in hapten-induced CHS responses (78). MCs represent a potential source of both pre-formed and *de novo* synthesized TNF (79–82). Biedermann and colleagues first demonstrated the importance of MC-derived TNF in CHS by showing that MC-deficient *Kit^W/W-v^* mice engrafted locally with *TNF*^−/−^ BMCMCs have reduced ear swelling and neutrophil recruitment in the skin following challenge with the hapten TNCB as compared to *Kit^W/W-v^* mice engrafted with WT BMCMCs (57). Using a similar approach in *Kit^W-sh/W-sh^* mice, it was latter demonstrated that MC-derived TNF is also required for optimal expression of oxazolone- and FITC-induced CHS (83, 84). In these studies, the authors reported that MCs and MC-derived TNF can promote nerve fiber elongation in the epidermis and dermis (83) as well as DC migration to the DLNs (84). More recently, the importance of MC-derived TNF in CHS was ascertained using *Mcpt5-Cre^+^; TNF^fl/fl^*, in which TNF is deleted specifically in connective tissue MCs (59). In this elegant study, the authors first used single-cell PCR in FACS-sorted peritoneal cells to demonstrate that the *Tnf* gene was efficiently deleted in MCs, but not in B cells and macrophages from *Mcpt5-Cre^+^; TNF^fl/fl^* mice (59). They then confirmed the role of MC-derived TNF in CHS by showing that *Mcpt5-Cre^+^; TNF^fl/fl^* mice have reduced ear swelling, as well as neutrophil and CD8^+^ T cell infiltration in the skin, following hapten challenge in a model of moderate DNFB-induced CHS (59). Confirming previous findings by Suto et al. (84), *Mcpt5-Cre^+^; TNF^fl/fl^* mice also had reduced migration of DCs from inflamed skin to skin-DLNs as compared to their littermate controls in this CHS model (84).

Besides TNF, MCs can produce and release a variety of pro-inflammatory factors which might contribute to CHS responses. Among these, immunohistochemistry data indicate that skin MCs produce CXCL2 (MIP-2) during CHS responses, and it was further reported that CXCL2 (MIP-2) blockade markedly reduces ear swelling and inflammation in this model (57). MC-deficient *Kit^W/W-v^* mice had reduced levels of CXCL2 in the skin as compared to *Kit^+/+^* littermates in this CHS model, and intradermal engraftment of BMCMCs into *Kit^W/W-v^* mice restored CXCL2 levels to those observed in *Kit^+/+^* mice (57). Altogether, these data suggest that MC-derived CXCL2 can play an important role in CHS, but formal demonstration of this will require more direct evidence using mice in which only MCs are deficient for this chemokine.

Skin MCs might also contribute to CHS through their ability to release histamine. However, acute CHS responses to the hapten TNCB were identical between WT mice and mice deficient for histidine decarboxylase (HDC) (85, 86), the enzyme responsible for synthesis of endogenous histamine from histidine in mammals. By contrast, the authors observed slightly reduced epidermal hyperplasia and leukocyte infiltration in *HDC*^−/−^ mice as compared to WT controls upon repeated challenges with TNCB, suggesting that histamine might contribute to these features in more chronic models of CHS (86). However, it should be noted that: (1) HDC-deficient mice have multiple phenotypic abnormalities, including decreased MC numbers and altered storage of various proteases in MC granules (87, 88) and (2) while MCs likely represent the major source of histamine in the skin, histamine can also be produced by other cell types, including basophils (89) and neutrophils (90, 91).

## Protective Functions of MCs in Severe and Chronic CHS Responses

Two studies performed in *Kit* mutant mice indicated that MCs can reduce the ear swelling, leukocyte recruitment, and tissue damage in models of severe acute CHS responses induced by either DNFB or oxazolone (60, 61) (Figure 2C). The mechanism of such protective effects was studied by Grimbaldeston et al., who showed that adoptive transfer of WT, but not *IL-10*^−/−^, BMCMCs into MC-deficient *Kit*-mutant mice reversed the exaggerated ear swelling and skin pathology (61). These results suggested that, in the model and experimental conditions tested, the enhanced CHS responses are due to the absence of MCs and MC-derived IL-10 in the *Kit*-mutant mice. However, these experiments did not prove that the same phenotypic abnormalities would necessarily be produced if MC-derived IL-10 was removed from the WT mice.

**Figure 3:**
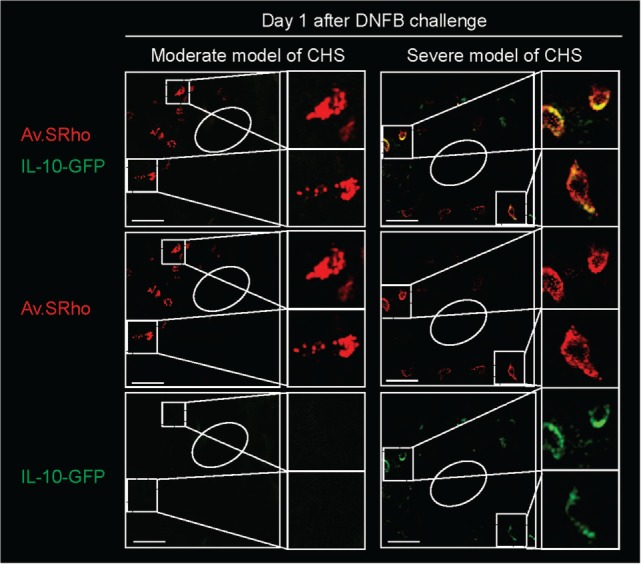
Skin mast cells (MCs) represent an early source of IL-10 during severe contact hypersensitivity (CHS) responses. We used Av.SRho in IL-10-GFP reporter mice to preform a longitudinal monitoring of both the release of dermal MC granules and activation of *Il10* gene transcription (IL-10-GFP, as detected by emission of GFP fluorescent signal) at the site of severe CHS responses using intravital 2-photon microscopy. Images show representative 3D photographs of the ear pinna one at day 1 upon DNFB challenge. Upper panel: merged fluorescence of Av.SRho (red) and IL-10-GFP (green). Middle panel: Av.SRho (red) fluorescence. Lower panel: IL-10-GFP (green) fluorescence. White dashed lines identify the magnified areas and white circles identify hair follicles (Scale bars: 50 µm). Adapted from Reber et al. (39).

We, therefore, recently decided to use a different approach to: (1) investigate whether MCs indeed represent a significant source of IL-10 during severe CHS responses and (2) determine whether deletion of IL-10 from endogenous MCs in non-*Kit* mutant mice could limit skin pathology during severe CHS responses. We used fluorochrome-labeled avidin in IL-10-GFP reporter mice, in which the GFP is placed under the control of the *il10* gene promotor, to simultaneously monitor MC granule release and MC *il10* gene activation in severe versus moderate models of DNFB-induced CHS (39). In the severe model of CHS, we found that dermal MCs released intracellular granule content into the surrounding microenvironment a few hours after induction of the pathology and that MCs represented one of the first immune cells to exhibit *Il10* gene activation at such sites of inflammation (Figure 3). Finally, using *Mcpt5-Cre^+^; Il10^fl/fl^* mice, in which the *il10* gene is specifically ablated in MCs, we confirmed that MC-derived IL-10 can significantly limit both the inflammation and the tissue pathology observed in severe CHS reactions (39) (Figure 2C).

However, we found that the enhancement of both the tissue swelling and the epidermal thickness associated with the reactions observed in *Mcpt5-Cre^+^; Il10^fl/fl^* mice was less pronounced than those observed in the *Kit*-independent MC-deficient mice. This suggested that MCs might help to limit these features of this acute model of severe CHS by both IL-10-dependent and IL-10-independent mechanisms. In contrast to the data obtained in the severe CHS model, in a more moderate CHS model we did not find evidence of *il10* gene activation in dermal MCs (39). These data align with previous results obtained by Dudeck et al. reporting statistically undistinguishable ear swelling in controls and *Mcpt5-Cre^+^; Il10^fl/fl^* mice in a similar moderate model of CHS (24).

The mechanism by which MCs can produce IL-10 during severe CHS responses was further assessed by Grimbaldeston and colleagues (61). It was reported that, when transferred intravenously into naïve recipient mice before sensitization, purified fractions of IgG obtained from mice immunized with urushiol can suppress CHS responses to the same hapten (92). The authors hypothesized that IgG immune complexes could suppress CHS responses, at least in part, by promoting production of IL-10 in MCs. Indeed, mouse MCs express the activating IgG receptor FcγRIII, as well as the inhibitory receptor FcγRIIB (93). Grimbaldeston et al. showed that IgG immune complexes can induce release of IL-10 in mouse BMCMCs *in vitro*. They also demonstrated that administration of an antigen-specific IgG1 antibody can suppress many features of the hapten-induced cutaneous responses in a model of passive transfer of CHS reactivity to *Rag2*^−/−^ mice (61). Finally, they reported that CHS responses in MC-deficient *Kit^W/W-v^* mice could be reduced to levels observed in *Kit^+/+^* mice after engraftment with WT BMCMCs, but not after engraftment with BMCMCs derived from *FcγRIII*^−/−^ or *FcRγ*^−/−^ mice (FcRγ is the common γ-chain shared by the high-affinity IgE receptor FcεRI and all IgG activating Fc receptors) (61). Altogether, these results suggest that IgG-induced aggregation of FcγRIII in MCs induces IL-10 production and is important for MC anti-inflammatory function during severe acute CHS responses (Figure 2C).

Most of the data on the role of MCs in CHS were obtained using acute models. However, chronic allergic contact dermatitis (CACD) is a chronic disease resulting from recurrent allergic skin inflammation triggered by episodic skin contact with relevant allergens (63). CACD might thus be better mimicked using more chronic models of CHS, in which mice are repeatedly challenged with haptens over the course of several days or weeks. Hershko et al. developed a model of chronic CHS consisting of sensitization with oxazolone on the ear followed in 1 week by the first of repeated challenges of the ear three times per week for up to a total of ten challenges (62). In this model, ear swelling was markedly increased in MC-deficient *Kit^W-sh/W-sh^* mice compared to their WT counterparts. Adoptive transfer of WT but not *IL-2*^−/−^ BMCMCs into *Kit^W-sh/W-sh^* mice dampened the inflammatory response, suggesting that MC production of IL-2 could suppress chronic CHS responses (62).

Interestingly, the authors observed similar results using *Kit^W-sh/W-sh^* mice engrafted with BMCMCs intravenously, a protocol which does not efficiently restore the MC population into the ear pinna (18, 62). These findings argue that IL-2 production by MCs located at sites distal from the ear could help reduce CHS responses in this chronic model. Using local intradermal engraftment with BMCMCs expressing the fluorescent GFP protein, the authors found that MCs may migrate from the injection site in the ear to the DLNs and then to the spleen (62). Increased MC numbers were also observed in this model in the spleen of WT mice, suggesting that endogenous MCs could also potentially migrate to the spleen in this chronic CHS model. Interestingly, such accumulation of MCs in the spleen was not observed in *Igh-7*^−/−^ mice, which lack the heavy chain of IgE antibodies (62). Moreover, and similar to the results obtained with MC-deficient mice, oxazolone challenge of *Igh-7*^−/−^ mice resulted in more severe eczematous dermatitis than was seen in *Igh-7^+/+^* mice (62). Finally, the authors reported that in the absence of MC IL-2 production, the ratio of activated to regulatory T cells (Treg) at the site of inflammation was increased. They thus suggested that in this chronic CHS model the spleen could serve as a site were MCs are recruited in an IgE-dependent manner and then produce IL-2, which maintains the effector T cells:Treg ratio at the site of inflammation and controls the severity of the disease (62) (Figure 2C). The immunosuppressive functions of Treg in CHS reactions have been extensively reviewed (94, 95). Interestingly, it was reported that Treg can directly suppress MC degranulation through cell–cell contact involving the OX40–OX40L interaction (96). Although this mechanism was not studied directly in the context of CHS responses, it is possible that such direct interactions might also regulate MC functions during CHS responses.

More recently, Gimenez-Rivera et al. developed a chronic CHS model consisting of sensitization with oxazolone followed by once-monthly challenges with the hapten for 3 or 4 months (63). Using this model, they observed more pronounced ear swelling in both *Kit^W-sh/W-sh^* mice and DT-treated *Mcpt5-Cre^+^; iDTR* MC-deficient mice as compared to their respective controls. Moreover, adoptive transfer of BMCMCs into *Kit^W-sh/W-sh^* mice restored responses to levels observed in WT mice (63). This study did not directly assess whether, similar to the findings of Hershko and colleagues (62), MCs in the spleen can also suppress CHS responses in this chronic model. However, the authors did report that MC-deficient mice have CHS responses comparable to those of the WT control mice at skin sites that had not been previously challenged with oxazolone, but that the MC-deficient mice exhibited exacerbated CHS responses in repeatedly challenged skin sites. They, therefore, concluded that local, and not systemic, effects are critical for the immunosuppressive effects of MCs in this chronic model (62, 63). Levels of Th1 cytokines and accumulation of antigen-specific IFN-γ-producing CD8^+^ T_RM_ cells were increased at skin sites chronically exposed to oxazolone in MC-deficient *Kit^W-sh/W-sh^* mice, as compared to *Kit^+/+^* mice. In addition, depletion of CD8^+^ T cells using an anti-CD8 depleting antibody reduced ear swelling in *Kit^W-sh/W-sh^* mice to levels observed in *Kit^+/+^* mice. Altogether, the authors concluded from these data that MCs can interfere with the exacerbated allergic skin inflammation in this chronic CHS model, at least in part, *via* effects on CD8^+^ T_RM_ cells (63).

## Conclusion and Future Perspectives

In this article, we have reviewed evidence that MCs can either limit or contribute to the inflammation and tissue pathology of CHS depending on the severity of the model used. It is tempting to speculate that, depending on their activation threshold, MCs could either become pro-inflammatory, i.e., “type 1” or inflammatory MCs, or anti-inflammatory, i.e., “type 2” or immunoregulatory MCs, as previously reported for other immune cells, such as macrophages (49). Whether these different functions of MCs can occur in settings other than CHS responses and the extent to which they reflect distinct MC phenotypes within the same population or different MC sub-populations expressing specific phenotypes and transcriptional programs remains to be investigated. Although care should be taken in extrapolating results obtained in mice to humans, our findings raise the possibility that immunoregulatory MCs might also help to dampen tissue inflammation associated with human contact dermatitis.

Inflammatory reactions, such as observed in models of CHS, are usually associated with complex and rapidly evolving biological processes. We have reviewed herein a broad panel of innovative genetic and imaging tools to investigate, and to directly visualize, the roles played by MCs in different mouse models mimicking the human responses (Table 1). The use of the Cre/LoxP approach is a particularly powerful tool to either deplete MC populations or delete selected genes specifically in MCs. This permits investigation of the roles of the MC, and MC expression of defined molecular targets, during disease development. Moreover, we have introduced the use of fluorochrome-labeled avidin as a non-genetic approach to probe and monitor skin MC granules. This offers a new alternative to the more time-consuming and expensive double transgenic approach and permits the unambiguous investigation of both MC gene activation and degranulation by intravital imaging in living mice in a genetically untouched environment.

We have focused our review on available data obtained using mouse models, but it goes without saying that one should always be cautious when extrapolating to humans the results obtained in mice. Clearly, there are likely to be multiple differences in both immune responses and disease pathogenesis in the two species. However, we propose that studies using newly described mouse models, in which MCs and their products can be monitored and manipulated, promise to reveal previously unsuspected roles of MCs in health and disease.

## Author Contributions

All authors were involved in drafting this review, and all authors approved the final version of the article.

## Conflict of Interest Statement

The authors declare that the research was conducted in the absence of any commercial or financial relationships that could be construed as a potential conflict of interest. The reviewer HS and handling Editor declared their shared affiliation.
